# Vaccination against *Clostridium difficile* by Use of an Attenuated *Salmonella enterica* Serovar Typhimurium Vector (YS1646) Protects Mice from Lethal Challenge

**DOI:** 10.1128/IAI.00089-19

**Published:** 2019-07-23

**Authors:** Kaitlin Winter, Li Xing, Audrey Kassardjian, Brian J. Ward

**Affiliations:** a Department of Microbiology and Immunology, McGill University, Montreal, Quebec, Canada; b Research Institute of the McGill University Health Centre, Montreal, Quebec, Canada; University of Michigan—Ann Arbor

**Keywords:** *Clostridium difficile*, toxin A, toxin B, YS1646, attenuated *Salmonella enterica* serovar Typhimurium, lethal challenge, vaccine

## Abstract

Clostridium difficile disease is mediated primarily by toxins A and B (TcdA and TcdB, respectively). The receptor binding domains (RBD) of TcdA and TcdB are immunogenic, and anti-RBD antibodies are protective. Since these toxins act locally, an optimal C. difficile vaccine would generate both systemic and mucosal responses. We have repurposed an attenuated Salmonella enterica serovar Typhimurium strain (YS1646) to produce such a vaccine. Plasmid-based candidates expressing either the TcdA or TcdB RBD were screened. Different vaccine routes and schedules were tested to achieve detectable serum and mucosal antibody titers in C57BL/6J mice. When given in a multimodality schedule over 1 week (intramuscularly and orally [p.o.] on day 0 and p.o. on days 2 and 4), several candidates provided 100% protection against lethal challenge. Substantial protection (82%) was achieved with combined p.o. TcdA and TcdB vaccination alone (days 0, 2, and 4). These data demonstrate the potential of the YS1646-based vaccines for C. difficile and strongly support their further development.

## Abstract (precis)

Clostridium difficile disease is mediated primarily by toxins A and B (TcdA and TcdB, respectively). The receptor binding domains (RBD) of TcdA and TcdB are immunogenic, and anti-RBD antibodies are protective. Since these toxins act locally, an optimal C. difficile vaccine would generate both systemic and mucosal responses. We have repurposed an attenuated Salmonella enterica serovar Typhimurium strain (YS1646) to produce such a vaccine. Plasmid-based candidates expressing either the TcdA or TcdB RBD were screened.

## INTRODUCTION

Clostridium difficile is one of the most important nosocomial pathogens in the world (1, 2). Clinically apparent C. difficile infection (CDI) is most often caused by antibiotics that disrupt the gastrointestinal microbiota, permitting the overgrowth of C. difficile and the production of toxins A and B (TcdA and TcdB, respectively). TcdA, an enterotoxin, and TcdB, a cytotoxin, represent two of the principal virulence factors of C. difficile (3), and both are expressed by most clinical isolates. Together, they disrupt the actin cytoskeleton of enterocytes in the gastrointestinal epithelium, resulting in fluid accumulation, inflammation, and severe tissue damage (4). Some strains of C. difficile produce an additional toxin called the binary toxin or CDT (5).

The prevalence and severity of CDI have increased significantly in most countries over the past 2 to 3 decades (2, 6). More than 370,000 cases occur every year in North America alone, with an estimated total cost exceeding $6 billion (7). Currently, antibiotics are routinely recommended for the treatment of CDI (e.g., metronidazole, vancomycin, and fidaxomicin alone or in combination), despite the irony of treating a disease caused by antibiotics with further antibiotics. Recurrent CDI after treatment and severe CDI are significant problems that are poorly responsive to antibiotics (8). Effective control of CDI is complicated by asymptomatic carriage, including asymptomatic carriage after treatment, and by spores that can persist in the environment for prolonged periods.

Preventing CDI-associated morbidity and mortality requires new approaches, including the development of vaccines. Clostridium difficile is noninvasive, so CDI is largely a toxin-mediated disease. Indeed, the outcome of CDI in both animal models and humans is strongly correlated with the host antibody response to TcdA and/or TcdB (9). These toxins have therefore been a major focus of both active and passive immunotherapeutic strategies, and several toxin-based vaccines have advanced to phase II/III clinical trials (10). Of particular interest to the current studies, both preclinical work (11, 12) and clinical-stage work (13) support the idea of targeting the receptor binding domains (RBDs) of these toxins. Whether whole protein, toxoid, or RBD is used, however, most of the effort to elicit antitoxin responses has focused on the peripheral, intramuscular (i.m.) administration of these antigens. Furthermore, as is typical for nonliving vaccines, these candidates require an adjuvant and multiple doses over several months to achieve an adequate immune response (10).

Several groups have demonstrated the potential of oral vaccines to elicit protective responses to RBDs in animal models of CDI. For example, Guo et al. demonstrated that oral administration of a Lactococcus lactis strain expressing both the RBDs of TcdA and TcdB can elicit both IgA and IgG and protect mice from lethal challenge (14). In conceptually similar studies, Hong and colleagues showed that hamsters given Bacillus subtilis spores expressing the carboxy-terminal segment of TcdA orally (TcdA_26–39_) can be protected from C. difficile colonization by mucosal IgA (15). We considered that a locally invasive but highly attenuated Salmonella enterica serovar Typhimurium vector might be even more effective in the induction of local and systemic anti-RBD responses. The flagellin protein of *S*. Typhimurium has been proposed to be a general mucosal adjuvant through its action on Toll-like receptor 5 (TLR5) (16). Ghose and colleagues have shown that the *S*. Typhimurium flagellin protein (Flic) fused to TcdA or TcdB can elicit toxin-specific IgA and IgG and protect mice from lethal challenge (17). Other *Salmonella* products, such as lipopolysaccharide (LPS), would be expected to further enhance immune responses by triggering additional pathogen recognition receptors (PRRs; e.g., TLR4) (18). Live attenuated *Salmonella* strains have other potential advantages as vaccine vectors, including targeting of the intestinal M cells that overlie the gut-associated lymphoid tissues (GALT) (19) and invasion of macrophages, leading to the induction of both humoral and cellular responses to their foreign protein cargo (20). They also have a large carrying capacity and are easy to manipulate both in the laboratory and at an industrial scale.

In recent years, live attenuated *Salmonella* has increasingly been used to express foreign antigens against infectious diseases and cancers (21–23). Salmonella enterica is a facultative intracellular pathogen that replicates in a unique membrane-bound host cell compartment, the *Salmonella*-containing vacuole (12). Although this location limits the exposure of both *Salmonella* and foreign proteins produced by the bacterium to the immune system, the organism’s type III secretion systems (T3SSs) can be exploited to translocate heterologous antigens into the host cell cytoplasm. Salmonella enterica encodes two distinct T3SSs within *Salmonella* pathogenicity islands 1 and 2 (SPI-I and SPI-II, respectively) that become active at different phases of infection (24). The SPI-I T3SS translocates effector proteins upon first contact of the bacterium with epithelium cells through to the stage of early cell invasion. In contrast, SPI-II expression is induced when the bacterium has been phagocytosed. Several effector proteins translocated by these T3SSs have been tested in the promotion of heterologous antigen expression in *Salmonella*-based vaccine development programs (23, 25), but how the effector protein-mediated secretion of heterologous antigens affects immune responses is still poorly understood. Although there is considerable experience in using the attenuated *S*. Typhi vaccine strain (Ty21a; Vivotif) in the delivery of heterologous antigens (22), we chose to use *S*. Typhimurium YS1646 as our candidate vector. This strain, originally named VNP20009, is attenuated by mutations in its *msbB* (LPS) and *purI* (purine biosynthesis pathway) genes and was originally developed as a tumor-targeting vector (26). With a major investment from Vion Inc., YS1646 was carried through preclinical and toxicity testing in rodents, dogs, and nonhuman primates before a phase I clinical trial, where it ultimately failed (27). More recently, YS1646 has been used to express a chimeric Schistosoma japonicum antigen in a murine model of schistosomiasis (28). Repeated oral administration of one of the engineered strains elicited a strong systemic IgG antibody response, induced antigen-specific T cells, and provided up to 75% protection against S. japonicum challenge.

In the current work, we exploited constitutive promoters and T3SS-specific promoters and secretory signals to generate 15 YS1646 strains with plasmid-based expression of the RBD portion of either TcdA or TcdB. These strains were screened for protein expression in monomicrobial culture and RAW 264.7 murine macrophages. The most promising constructs were advanced to immunogenicity testing in adult female C57BL/6 mice using different routes (e.g., recombinant protein i.m., YS1646 strains orally [p.o.]) and schedules (e.g., repeat dosing, multimodality, prime-pull) to achieve the best serologic response in the shortest period of time. Two of the YS1646 strains elicited strong systemic IgG responses and provided up to 100% protection from lethal challenge when administered in a multimodality schedule over 5 days (i.m. and p.o. on day 0 followed by p.o. boosting on days 2 and 4).

## RESULTS

### Transformed *S*. Typhimurium YS1646 expresses heterologous antigen.

**FIG 1:**
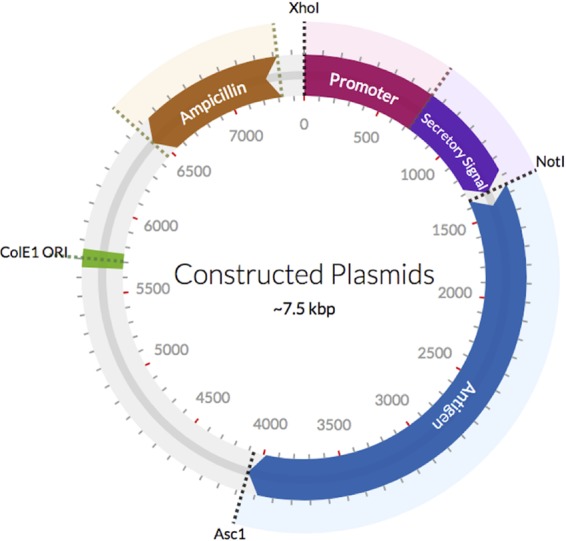
Generic plasmid map. The pQE_30 plasmid containing an ampicillin resistance gene was used as the backbone. The promoter and secretory signals were inserted between XhoI and NotI sites. The antigen sequence was inserted between NotI and AscI sites. Plasmids were between 3.4 kbp (pQE_null) and 7.5 kbp in size.

Plasmids expressing the RBDs of toxin A (rbdA) or toxin B (rbdB) under the control of different promoters and secretory signals were constructed (Fig. 1). The promoter-secretory signal combinations included SPI-I-specific (e.g., SopE2, SptP) and SPI-II-specific (e.g., SseJ, SspH2) pairings as well as pairings used by both the SPI-I and SPI-II secretory pathways (e.g., SteA, SteB, SspH1). Some of the secretory signals were also paired with the constitutively active or inducible promoters *nirB*, *pagC*, and *lac* (Table 1). All primers used in the study are listed in Table S1 in the supplemental material. A set of plasmids with the same promoter/secretory signal pairings but expressing enhanced green fluorescent protein (EGFP) was also constructed. All plasmids were transformed into *S*. Typhimurium YS1646.

**TABLE 1:** Plasmids used in this study

Plasmid	Promoter	Secretory signal	Antigen
pQE_null			
pSopE2_SopE2_rbdB	*sopE2*	SopE2	TcdB_1821–2366_
pSseJ_SseJ_rbdB	*sseJ*	SseJ	TcdB_1821–2366_
pSptP_SptP_rbdB	*sptP*	SptP	TcdB_1821–2366_
pSspH1_SspH1_rbdB	*sspH1*	SspH1	TcdB_1821–2366_
pSspH2_SspH2_rbdB	*sspH2*	SspH2	TcdB_1821–2366_
pSteA_SteA_rbdB	*steA*	SteA	TcdB_1821–2366_
pSteB_SteB_rbdB	*steB*	SteB	TcdB_1821–2366_
ppagC_SspH1_rbdB	*pagC*	SspH1	TcdB_1821–2366_
pSspH2_SspH2_rbdA	*sspH2*	SspH2	TcdA_1820–2710_
plac_SopE2_rbdA	*lac*	SopE2	TcdA_1820–2710_
plac_SspH1_rbdA	*lac*	SspH1	TcdA_1820–2710_
pnirB_SopE2_rbdA	*nirB*	SopE2	TcdA_1820–2710_
pnirB_SspH1_rbdA	*nirB*	SspH1	TcdA_1820–2710_
ppagC_SopE2_rbdA	*pagC*	SopE2	TcdA_1820–2710_
ppagC_SspH1_rbdA	*pagC*	SspH1	TcdA_1820–2710_

**FIG 2:**
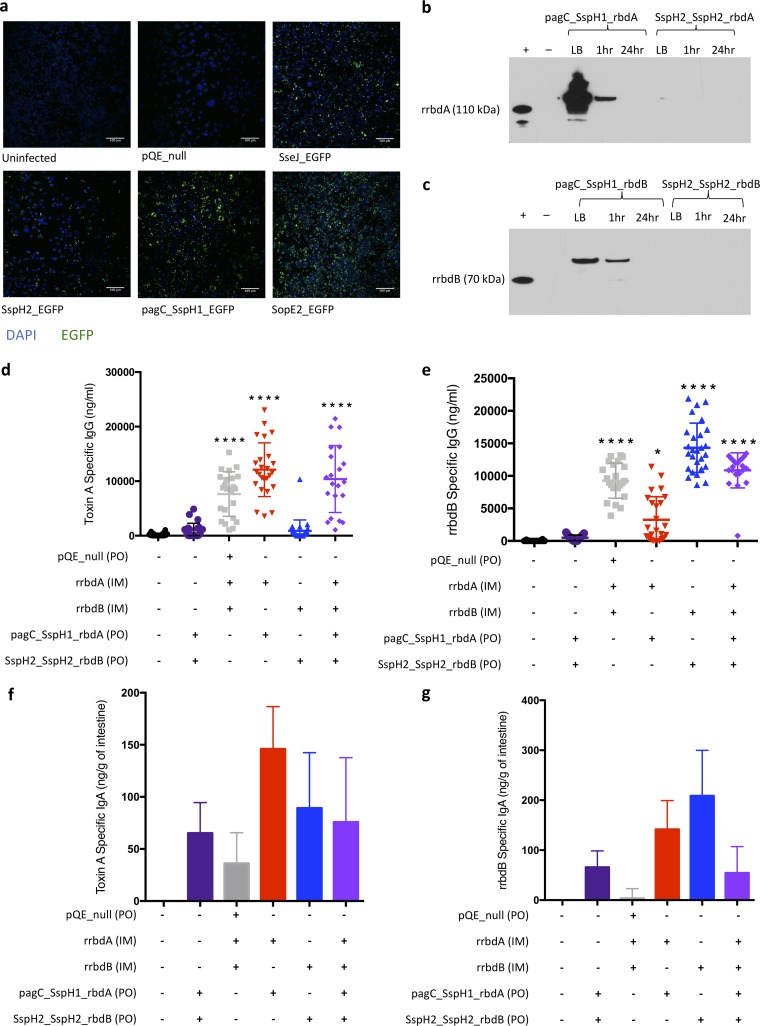
Transformed YS1646 strains expressed heterologous antigen. (a) EGFP-expressing strains of YS1646 were added to RAW 264.7 macrophages *in vitro* and visualized 24 h later using a fluorescence microscope. Images are representative of two repeats. (b and c) Antigen expression was examined by Western blotting from YS1646 strains transformed with rbdA (b) and rbdB (c) plasmids. Samples were collected after 16 h of growth in LB and 1 h and 24 h after infection of RAW 264.7 macrophages. Gels were run with a positive control (recombinant RBD antigen, without secretion signals, produced in E. coli), and the film was exposed for 2 min. The increased sizes of the RBDs produced in YS1646 are consistent with the secretion signals that were not cleaved. Mice were immunized with a dose of 10 μg recombinant antigen (rrbdA and/or rrbdB) intramuscularly and three doses of 1 × 10^9^ CFU of antigen expressing YS1646 (pagC_SspH1_rbdA and/or SspH2_SspH2_rbdB) orally every other day. (d and e) Serum was collected at 3 to 4 weeks after vaccination, and toxin A-specific IgG (d) and rrbdB-specific IgG (e) were detected by ELISA (*n* = 21 to 28, 4 repeats). Data are presented as the mean and standard deviation (SD). (f and g) Intestines were collected 5 weeks after vaccination, and toxin A-specific IgA (f) and rrbdB-specific IgA (g) were detected by ELISA (*n* = 4 to 5, one repeat). Data are presented as the mean and standard error of the mean (SEM) value from which the mean of the PBS control was subtracted. The Kruskal-Wallis test and Dunn’s multiple-comparison test were used to compare all groups. All *P* values are by comparison to the PBS control group. *, *P* < 0.05; ****, *P* < 0.0001.

Using the EGFP-expressing strains, we screened for antigen expression in monomicrobial culture and during *in vitro* infection of murine RAW 264.7 macrophages. Most strains produced detectable EGFP in monomicrobial culture (summarized in Table S2). The YS1646 candidates were readily macropinocytosed, and a fluorescent signal was detected for all of the EGFP-expressing strains (Fig. 2a). Expression varied considerably between strains, with the strongest signal being driven by the pagC_SspH1_EGFP construct. Some constructs (e.g., SspH2_SspH2_EGFP) had good initial EGFP expression, but survival and/or replication in the macrophages was markedly reduced at 24 h postinfection.

Expression of the targeted C. difficile RBDs in monomicrobial culture and murine macrophages was examined by Western blotting at 1 and 24 h postinfection. Modest production of rbdA and rbdB could be documented by most strains in monomicrobial culture, but very few strains had detectable antigen expression during macrophage infection (rbdA in Fig. 2b and rbdB in Fig. 2c; summarized in Table S2). For example, the pagC_SspH1 pairing drove strong expression of both antigens in broth and at 1 h postinfection in the murine macrophages, but the SspH2_SspH2 pairing failed to drive detectable rbdB expression, and the level of rbdA production was barely detectable only in monomicrobial culture. Secretion of the RBDs into extracellular medium was examined in monomicrobial culture (Table S2). Only pagC_SspH1_rbdA had detectable antigen secretion. The lack of secretion detection may have been due to low levels of expression in the cells.

The most promising constructs were advanced to mouse immunogenicity testing. Since neither monomicrobial culture nor RAW 264.7 cells are adequate models for the low-oxygen-tension and polymicrobial environment of the gastrointestinal tract, we included some of the apparently negative constructs in the *in vivo* immunogenicity testing.

### rbdA and rbdB delivered by YS1646 in combination with recombinant rbdA and rbdB are highly immunogenic in mice.

Using the rapid induction of serum antigen-specific IgG as our principal screening tool, a multimodal schedule was identified to be the most promising vaccination strategy. This schedule was comprised of a single i.m. dose of the recombinant RBD (rrbd) on day 0 with 3 p.o. doses of the corresponding RBD-expressing strain on days 0, 2, and 4. When sera were collected 3 to 4 weeks after vaccination using this schedule, rbdA-specific (Fig. 2d) and rbdB-specific (Fig. 2e) IgG titers were consistently elevated. The IgG responses generated were consistently higher than those achieved by recombinant antigen delivered i.m. and the pQE_null strain delivered p.o., but these differences did not reach statistical significance (*P* = 0.1727 for rbdB). In contrast, mice that received only the three p.o. doses of YS1646 strains bearing the RBD antigens had no detectable serum IgG response. Despite the failure to induce IgG with p.o. vaccination, three doses of YS1646 on alternate days could nonetheless prime for a significant response to a subsequent i.m. booster dose delivered 3 weeks later (data not shown). Both the multimodal and oral-only vaccination schedules generated higher rbdA-specific IgA (Fig. 2f) and rbdB-specific IgA (Fig. 2g) levels in the intestinal tissues than delivery of the recombinant antigen intramuscularly, although the differences did not reach statistical significance with the relatively small number of animals used in these experiments. Interestingly, mice vaccinated against only one toxin tended to have higher IgA antibody titers against that toxin than mice vaccinated against both toxins, raising the possibility of some degree of antigen interference.

### Selection of candidate YS1646 strains for challenge testing.

The combined screening studies identified two YS1646 constructs that were carried forward into challenge testing (pagC_SspH1_rbdA and SspH2_SspH2_rbdB) (Table S2). Since oral immunization generated intestinal IgA (Fig. 2f and g) and was able to prime animals for a strong systemic IgG response to a subsequent i.m. boost (data not shown), we included p.o. only groups in challenge studies, in addition to the multimodality i.m. plus p.o. schedule.

### YS1646-vectored rbdA and rbdB vaccines protect mice from lethal C. difficile challenge.

**FIG 3:**
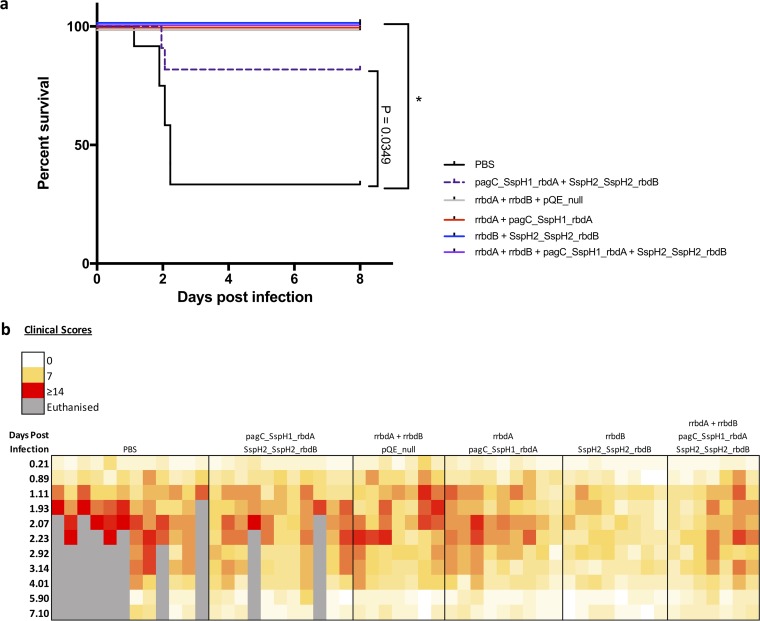
Vaccination with receptor binding domain (rbd) antigens protected against C. difficile challenge. Mice were immunized with a dose of 10 μg of recombinant antigen (rrbdA and/or rrbdB) intramuscularly and three doses of 1 × 10^9^ CFU of antigen expressing YS1646 (pagC_SspH1_rbdA and/or SspH2_SspH2_rbdB) orally every other day. At 5 weeks after vaccination, mice were challenged p.o. with freshly cultured C. difficile (1.97 × 10^5^ CFU and 1.70 × 10^7^ CFU). Mice were clinically scored 1 to 3 times daily by an observer blind to the treatment. A score of ≥14/20 and/or a >20% loss of the starting body weight was considered the humane endpoint. Survival (a) and clinical scores (b) are shown (*n* = 7 to 12, 2 repeats). The log-rank (Mantel-Cox) test was used to compare all groups to the PBS control group. Correction of the *P* value for multiple comparisons was done using the Bonferroni method. *, *P* < 0.01 compared to the PBS control group.

At 5 weeks after vaccination, mice were challenged with a lethal dose of C. difficile vegetative cells and monitored for weight loss, clinical score, and death. Overall, 67% of the phosphate-buffered saline (PBS)-treated control group succumbed to infection at between 36 and 72 h postinfection (Fig. 3a). Only 18% of the mice that received three p.o. doses of the pagC_SspH1_rbdA and SspH2_SspH2_rbdB strains succumbed to the infection. All other vaccinated groups had 100% survival (Fig. 3a). The recovery of animals that survived appeared to be complete: surviving mice recovered their original body weight. Mice were followed for up to 3 weeks after infection, and no relapses were observed. During infection, mice were clinically scored 1 to 3 times daily (Fig. 3b). Although the group vaccinated with rrbdA plus rrbdB i.m. and the pQE_null strain p.o. had 100% survival, 71% of these mice were severely ill, achieving a score of 12 or higher (a score of 14 was the animal care cutoff for the humane endpoint). The proportion of severely ill mice in groups that received any antigen-expressing YS1646 strain with an i.m. dose of recombinant protein was consistently much lower (0% to 14%). None of the animals in the group that received rrbdB i.m. plus three doses of the SspH2_Ssph2_rbdB strain p.o. experienced severe illness. All mice had very low or completely normal clinical scores by 6 days postinfection. There was a strong negative correlation between serum anti-rbdB IgG both before and after challenge and the highest clinical score achieved by individual mice (Fig. 2e; Fig. S1b and d; Table S3). Our results suggest that in our mouse model, an immune response directed toward TcdB is sufficient to obtain effective protection from C. difficile challenge.

The combined i.m. and p.o. schedules also elicited small but detectable increases in antigen-specific IgA levels in the intestinal tissues after challenge, although the increase reached statistical significance only for the animals vaccinated against rbdB alone (*P* < 0.05 versus the control group) (Fig. S1c and d). Interestingly, the intestinal anti-rbdB IgA levels tended to be slightly lower in the animals that received both of the YS1646 constructs p.o. than in those vaccinated only against rbdB (Fig. 2f; Fig. S1d), although this difference also failed to reach statistical significance.

## DISCUSSION

The pathology associated with CDI is thought to be toxin mediated (3), and there are strong precedents for the efficacy of vaccine-induced antitoxin antibodies in the prevention or modification of toxin-mediated diseases (e.g., tetanus, diphtheria, cholera) (29, 30). Indeed, an anti-TcdB monoclonal antibody (bezlotoxumab [Zinplava; Merck]) has recently been shown to reduce the frequency of recurrent C. difficile disease (31). In addition to passive immunotherapy, the generation of antitoxin antibodies is also the predominant strategy being pursued by both large and small pharmaceutical companies with an interest in developing C. difficile vaccines (10). However, the most advanced of these candidate vaccines require multiple doses of antigen with an adjuvant over several months to achieve high serum antibody concentrations (10, 32). Furthermore, even though CDI is a disease of the gastrointestinal mucosa, none of these candidates would be expected to generate an effective mucosal immune response. Both in theory and as demonstrated in the current work, the delivery of the same C. difficile toxin antigens using a live attenuated *S*. Typhimurium vector has the potential to induce both local and systemic immunity. There are several groups working on delivering C. difficile antigen at the mucosal surface (15, 33). Recently, Wang et al. used a nontoxigenic C. difficile strain to target TcdB and TcdA (33). They found that after 3 doses delivered every 2 weeks, their vaccine candidate was effective at protecting mice and hamsters. In this study, we provide a proof of concept that a multimodality vaccination schedule using a single i.m. dose of recombinant toxin A and/or toxin B receptor binding domain proteins with p.o. delivery of YS1646 bearing the same RBD antigens over a 5-day period can rapidly induce both systemic and mucosal responses and protect mice from an otherwise lethal challenge. Although the amount of IgA present in the intestinal tissues after vaccination was relatively low after YS1646 vaccination, the induction of an effective local immune response by these vaccines was strongly supported by the fact that oral vaccination alone provided substantial protection, despite the absence of detectable serum antibodies prior to challenge.

Although they are logistically more complicated and considered inelegant by some, heterologous prime-boost and multimodality vaccination strategies are gaining traction for a wide range of infections and other complex conditions, such as cancers (34–36). Of particular interest to the current proposal, such combined-modality approaches have shown promise in eliciting effective immune responses against mucosal pathogens, such as human immunodeficiency virus/simian-human immunodeficiency and influenza virus (35, 36). Combined-modality strategies may also have a place in toxin-mediated diseases in which high titers of preformed antibodies are needed, such as in Clostridium perfringens infection (37), or when a rapid but sustained response is desirable, such as in Ebola (38). These new approaches have the potential to enhance the character, kinetics, and durability of the response (39). While simpler vaccination strategies will certainly be carried forward as our candidate vaccines advance into larger-animal models (40, 41), toxicity testing, and, ultimately, clinical trials, the multimodality method that we developed in the murine model would be relatively easy to administer to the typical person who might benefit from a C. difficile vaccine, i.e., those in or entering a long-term-care facility (42) or being prepared for elective surgery. Only one face-to-face clinic/office visit would be needed to receive the i.m. vaccine and the first (supervised) p.o. vaccine, after which the remaining two p.o. doses on alternate days could be taken autonomously (as is currently the practice for the live attenuated *S*. Typhi Ty21a vaccine). The long clinical experience with Ty21a also confirms the feasibility of delivering attenuated *Salmonella* to the intestinal tissues (22). Although such a rapid vaccination schedule would likely increase compliance, it is also possible that the durability of the response would be compromised (43). Clearly, long-term follow-up studies will be needed to more completely evaluate the optimal vaccination strategy for the YS1646 vaccine candidates.

While still early in development, there are certainly safety concerns in potentially exposing elderly or debilitated individuals to a live attenuated bacterium as a vaccine vector. Several of the immunological and physiological factors that put the elderly at risk for C. difficile infection also make a live attenuated vaccine that targets the gut mucosa a potential risk. Even though wild-type *S*. Typhimurium typically causes only mild disease localized to the gastrointestinal tract in humans (22), it can sometimes cause invasive disease with serious outcomes (44). The YS1646 strain that is the backbone of our vaccine platform carries mutations of both an LPS gene (*msbB*) and a part of the purine production machinery (*purI*) that render it highly attenuated (27). Although the mechanisms of attenuation differ, the live attenuated Ty21a *S*. Typhi vaccine has an excellent safety record, even in elderly subjects (22). In the critical development pathway of YS1646 as a possible anticancer agent in the early 2000s, this strain proved to be safe in multiple small-animal models (e.g., mice, rats) and large-animal models (e.g., dogs, rhesus macaques) (D. Bermudes, unpublished data) before it was permitted to advance to a phase I clinical trial (27). In this trial, a single dose of up to 3 × 10^8^ CFU of YS1646 was administered intravenously to 24 subjects with metastatic melanoma or renal cell carcinoma without any major safety signals. Most of the subjects in this trial cleared YS1646 from their bloodstream in <12 h (27). It was subsequently suggested that an unexpected susceptibility of YS1646 to the physiologic levels of CO_2_ present in human tissues (∼5%) may have contributed to its failure as a cancer therapy (45). In contrast to the need for YS1646 to disseminate and replicate actively in tumor tissues as an anticancer agent, to be an effective vaccine vector, YS1646 needs to invade locally and express the targeted antigen in the GALT for only a short period of time (22). Of course, a necessary step prior to the use of YS1646 as a candidate C. difficile vaccine will be chromosomal integration of the most promising TcdA and TcdB RBD constructs; this work is under way. Although chromosomal integration will reduce the copy number of our target gene and, therefore, protein expression, we will try to mitigate these effects through the use of strong promoters (e.g., P*pagC*) and the integration of tandem repeats for both antigens. Since several of our current candidates were able to elicit immune responses, despite undetectable antigen production *in vitro*, we are optimistic that we will be able to design chromosomally integrated strains that are immunogenic. To our knowledge, the only other clinical experience with attenuated *S*. Typhimurium is that of Hindle et al., who exposed a small number of human subjects to a single oral dose of up to 1 × 10^9^ CFU of a strain bearing *aroC* and SPI-II T3SS mutations without dissemination or ill effects (46). Hindle et al. observed asymptomatic shedding of an attenuated *S*. Typhimurium strain for 3 weeks in the feces of patients, with all shedding ending by week 4 after vaccination (46). Although YS1646 has different attenuating mutations and may have a different colonization profile in humans after oral delivery, the asymptomatic persistence of this *S*. Typhimurium strain was also demonstrated for at least 1 week in a small proportion of subjects after intravenous delivery in the early anticancer phase I trial (27). While we acknowledge that the question of colonization/persistence will eventually need to be addressed with regulators should a YS1646-vectored C. difficile vaccine enter into clinical trials, the mere fact of persistence does not automatically disqualify a vaccine candidate. Indeed, several of the live attenuated vaccines on the market are routinely shed by vaccinees for longer than a week. These include the rotavirus vaccine, which is shed for up to 9 days postvaccination (47); the measles vaccine, which can be detected for at least 14 days (48); the oral polio vaccine, which can persist for several months (49); and the varicella vaccine, which causes a life-long latent infection (50).

This study has several limitations. First, there is no perfect small- or large-animal model for human CDI (40, 41). Although mice are widely considered to be one of the most informative models, mice are also the natural host for *S*. Typhimurium. Indeed, *S*. Typhimurium infection in mice is commonly used as a model for human typhoid fever caused by *S*. Typhi (51). As a result, the degree to which an attenuated *S*. Typhimurium strain, such as YS1646, will have a similar profile of attenuation in mice and humans is unknown. Indeed, although mice remained completely healthy during and after oral vaccination, we observed colonization of the spleen and liver by some of the YS1646 strains carrying either TcdA or TcdB constructs for 1 to 2 weeks after vaccination (data not shown). Although we do not expect to see such dissemination in humans due to the CO_2_ sensitivity of YS1646, it is certainly possible that the immunity generated in response to persistent antigen expression over days to weeks will differ from that induced by a shorter exposure. Such persistence may not occur in other models, such as the gnotobiotic piglet (40, 41), which has been used as a large-animal model for C. difficile infection. Second, the relative sensitivity of the different animals used in C. difficile studies and humans to the major C. difficile toxins is not fully consistent (52). Nonetheless, it is likely that both TcdA and TcdB contribute to pathology in the mouse model that we are using and in humans (53). As a result, we are optimistic that our findings in the murine model will predict outcomes in humans, and our goal is to develop a YS1646-based vaccine that can provide protection against both TcdA and TcdB. Finally, the choice of optimal promoter-secretory signal pairings for *in vivo* expression of the RBD antigens is complicated by our inability to truly reflect the conditions to which the YS1646 strains will be exposed in the human gastrointestinal tract and the GALT. We have tried to mitigate this risk by using a multilayered screening process but acknowledge that we have already identified constructs that do not appear to produce the targeted RBD *in vitro* (in monomicrobial culture or RAW 264.7 cells) but still elicit strong antibody responses in the mouse model.

In this work, we describe the repurposing of a live attenuated *S*. Typhimurium strain (YS1646) as a vaccine vector to target the major toxins of C. difficile. When administered in a 5-day, multimodality schedule (i.m. 1 time, p.o. 3 times), these candidate vaccines elicited high serum IgG titers and provided complete protection from lethal challenge in a mouse model. This proof-of-concept study supports the further development of these candidate vaccines by chromosomal integration of the two most promising constructs (SspH2_Ssph2_rbdB and pagC_SspH1_rbdA), evaluation in the gnotobiotic piglet model (40, 41), and toxicity testing. If these next steps are successful, a phase I human study with a mixed TcdA-TcdB vaccine will be pursued.

## MATERIALS AND METHODS

### Bacterial strains and growth conditions.

Salmonella enterica Typhimurium YS1646 (Δ*msbB2* Δ*purI* Δ*Suwwan xyl* negative; ATCC 202165; ATCC, Manassas, VA) was obtained from Cedarlane Labs (Burlington, ON, Canada). Escherichia coli DH5α (Thermo Fisher Scientific, Eugene, OR) was used for the production of recombinant plasmids. Plasmids were introduced into E. coli or YS1646 by electroporation (2 μg of plasmid at 3.0 kV, 200 Ω, and 25 μF; GenePulser XCell, Bio-Rad, Hercules, CA, USA). Transformed bacteria were grown in Luria broth (LB) with 50 μg/ml of ampicillin (Wisent, St. Bruno, QC, Canada) for cells containing plasmids with the pQE_30 backbone.

Clostridium difficile strain VPI 10463 (ATCC 43255) was obtained from Cedarlane Labs and used for the challenge experiments. Cells were maintained in meat broth (Sigma-Aldrich, St. Louis, MO) containing 0.1% (wt/vol) l-cysteine (Sigma-Aldrich) in an anaerobic jar. For colony counts, C. difficile-containing medium was serially diluted and streaked onto prereduced brain heart infusion (BHIS) plates (BD Biosciences, Mississauga, ON, Canada) containing 0.1% (wt/vol) l-cysteine. The bacteria were left to grow on the plates at 37°C in an anaerobic jar for 24 h.

### Plasmid construction. (i) Vaccine candidate plasmids.

The pQE_30 plasmid backbone containing an ampicillin resistance gene used for antigen expression in the vaccine candidates was cloned from the plasmid roGFP_IL_pQE30, a gift from David Ron (plasmid number 48633; Addgene) (54). PCR was used to obtain the SopE2, SptP, SseJ, SspH1, SspH2, SteA, and SteB promoter and secretory signal sequences from YS1646. The *pagC* promoter from YS1646 and the *nirB* promoter from E. coli were also PCR amplified. The *lac* promoter was incorporated into the 5′ PCR primer. The antigenic C-terminal ends of the receptor binding domains for toxin A (TcdA_1820–2710_) and toxin B (TcdB_1821–2366_) were amplified by PCR from C. difficile VPI 10463. Restriction sites were incorporated 5′ of the promoters (XhoI), between the secretory signal and the antigen (NotI), and at the 3′ end of the antigen sequence (AscI) (Fig. 1). The primers used are listed in Table S1 in the supplemental material. DNA sequencing confirmed that plasmids had the expected sequence (McGill University Genome Centre, Montreal, QC, Canada). The EGFP antigen was cloned from the plasmid pEGFP_C1 (Clontech, Mountain View, CA) with the NotI and AscI sites incorporated into the primers. All plasmids are named based on the promoter, secretory signal, and antigen used, and these are described in Table 1. The unedited pQE_30 plasmid was transformed into YS1646 as a control and is referred to as pQE_null.

### (ii) Recombinant TcdA and TcdB expression.

Protein expression and purification of recombinant TcdA_1820–2710_ (rbdA) and TcdB_1821–2366_ (rbdB) were accomplished using the pET-28b plasmid (Novagen, Millipore Sigma, Burlington, MA) with an isopropyl-β-d-1-thiogalactopyranoside (IPTG)-inducible promoter and kanamycin resistance gene. A 6× His tag and stop codon were added at the 3′ end. The expression vector was transformed into E. coli C2566l (New England BioLabs, Whitby, ON, Canada) as described above. Transformed bacteria were grown in a 37°C shaking incubator with 30 μg/ml of kanamycin (Wisent), until the optical density (absorbance) at 600 nm (OD_600_) reached 0.5 to 0.6. IPTG (Invitrogen, Carlsbad, CA) was then added, and expression was induced for 3 to 4 h. Cells were pelleted by centrifugation at 3,000 × *g* for 10 min at 4°C. The cells were lysed, and the lysate was collected and purified using Ni-nitrilotriacetic acid (NTA) affinity chromatography (Ni-NTA Superflow; Qiagen, Venlo, Limburg, Netherlands). The eluate was analyzed by Coomassie blue staining of polyacrylamide gels and Western blotting using a monoclonal antibody directed against the His tag (Sigma-Aldrich).

### Macrophage infection.

RAW 264.7 cells (ATCC TIB-71) were cultured in Dulbecco's modified Eagle's medium (DMEM; Wisent) supplemented with 10% fetal bovine serum (FBS), penicillin (100,000 U/ml), and streptomycin (100 μg/ml; Wisent); cells were passaged when they reached ∼90% confluence. For each passage, cells were washed with Hanks’ balanced salt solution (HBSS) without calcium and magnesium (Wisent) and detached from the flasks using 0.25% trypsin (Wisent). RAW 264.7 cells were seeded in Falcon polystyrene 12-well plates (Corning Inc., Corning, NY) at a density of 1 × 10^6^ cells/well for infection experiments 24 h later. RAW 264.7 cells were infected at a multiplicity of infection (MOI) of either 40 or 100. For Western blotting, cells were then incubated at 37°C in 0% CO_2_, as YS1646 is sensitive to increased CO_2_ levels. Infection was allowed to proceed for an hour, and then the cells were washed 3 times with PBS and resuspended in DMEM to which 50 μg/ml of gentamicin (Wisent) was added to kill extracellular YS1646. After 2 h, the gentamicin concentration was lowered to 5 μg/ml.

### (i) Fluorescence (EGFP) microscopy.

RAW 264.7 cells, plated on 8-well microscope chamber slides (Eppendorf, Hamburg, Germany) at 1.8 × 10^5^ cells/chamber, were infected at an MOI of 40 with YS1646 strains transformed with the EGFP constructs. Infected cells were incubated at 37°C in 5% CO_2_. At 24 h after infection, cells were stained with 4′,6-diamidino-2-phenylindole (DAPI; Thermo Fisher Scientific) and fixed with 4% paraformaldehyde (Sigma-Aldrich). A Zeiss LSM780 laser scanning confocal microscope was used for imaging (a 405-nm laser for excitation of DAPI, a 488-nm laser for excitation of EGFP), and acquisition and processing were performed using ZEN software (Zeiss, Toronto, ON, Canada).

### (ii) Western blotting.

For antigen expression in monomicrobial culture, the transformed YS1646 strains were grown overnight in LB with 50 μg/ml of ampicillin at 37°C in 0% CO_2_, centrifuged at 21,130 × *g* for 10 min, resuspended in PBS, and then mixed in with NuPAGE lithium dodecyl sulfate (LDS) sample buffer (Invitrogen) according to the manufacturer’s instructions. For antigen expression in RAW 264.7 macrophages, infection was allowed to proceed for either 1 h or 24 h. Samples were then collected, centrifuged, resuspended in PBS, and mixed with sample buffer as described above. All samples were heated for 10 min at 70°C and then cooled on ice. Proteins were separated on a 4 to 12% Bis-Tris protein gel (Invitrogen) and transferred to nitrocellulose membranes using a Trans-Blot Turbo RTA mini-nitrocellulose transfer kit (Bio-Rad, Hercules, CA). For detection of TcdA_5458–8130_ and TcdB_5461–7080_, the membranes were incubated first with anti-toxin A chicken IgY (1:5,000; Abnova, Taipei, Taiwan) and anti-toxin B chicken IgY (1:10,000; Abnova) antibodies, respectively, followed by goat anti-chicken IgY conjugated to horseradish peroxidase (1:10,000; Thermo Fisher Scientific). Immunoreactive bands were visualized using the SuperSignal West Pico Plus chemiluminescent substrate (Thermo Fisher Scientific) and autoradiography film (Denville Scientific, Holliston, MA).

### Mice.

Six- to 8-week-old female C57BL/6J mice were obtained from Charles River Laboratories (Montreal, QC, Canada) and were kept under pathogen-free conditions in the Animal Resource Division at the McGill University Health Center Research Institute (RI-MUHC). All animal procedures were approved by the Animal Care Committee of McGill University and performed in accordance with the guidelines of the Canadian Council on Animal Care.

### (i) Vaccination.

For oral vaccinations, mice were gavaged with 1 × 10^9^ CFU of the YS1646 strains in 0.2 ml of PBS (days 0, 2, and 4). When both strains were given, 5 × 10^8^ CFU of each strain was used, for a total of 1 × 10^9^ CFU of YS1646 given in 0.2 ml of PBS. Intramuscular (i.m.) injections contained a total of 10 μg of recombinant protein and 250 μg of aluminum hydroxide gel (alum; Alhydrogel; Brenntag BioSector A/S, Frederikssund, Denmark) in 50 μl, which was administered into the gastrocnemius muscle using a 28-gauge needle.

### (ii) Blood and intestine sampling.

Baseline serum samples were collected from the lateral saphenous vein prior to all other study procedures using Microtainer serum separator tubes (Sarstedt, Nümbrecht, Germany). Serum samples were also collected from the mice at the end of the study by cardiac puncture after isoflurane-CO_2_ euthanasia. Serum separation was performed according to the manufacturer’s instructions, and aliquots were stored at −20°C until they were used. At study termination, 10 cm of the small intestine, starting at the stomach, was collected. Intestinal contents were removed, and the tissue was weighed and stored in a protease inhibitor (PI) cocktail (catalog number P8340; Sigma-Aldrich) at a 1:5 (wt/vol) dilution on ice until it was processed. The tissue was homogenized (Homogenizer 150; Fisher Scientific, Ottawa, ON, Canada) and centrifuged at 2,500 × *g* at 4°C for 30 min, and the supernatant was collected. Supernatants were stored at −80°C until they were analyzed by enzyme-linked immunosorbent assay (ELISA). For postchallenge data, samples were collected from survivors at 3 weeks after infection.

### (iii) Clostridium difficile challenge.

C. difficile challenge experiments were performed essentially as described previously (55, 56). Briefly, mice were preadapted to acidic water by adding acetic acid at a concentration of 2.15 μl/ml (vol/vol) to their drinking water 1 week prior to antibiotic treatments. At 6 days prior to infection, an antibiotic cocktail that included metronidazole (0.215 mg/ml; Sigma-Aldrich), gentamicin (0.035 mg/ml; Wisent), vancomycin (0.045 mg/ml; Sigma-Aldrich), kanamycin (0.400 mg/ml; Wisent), and colistin (0.042 mg/ml; Sigma-Aldrich) was added to the drinking water. After 3 days, regular water was returned, and at 24 h prior to infection, mice received clindamycin (32 mg/kg of body weight; Sigma-Aldrich) intraperitoneally in 0.2 ml of PBS using a 28-gauge needle. Fresh C. difficile cultures were used in our challenge model so that the dose used was estimated on the day of infection based on OD_600_ values and the precise inoculum was calculated 24 h later. This procedure led to the use of different C. difficile doses in the two challenge studies performed (1.7 × 10^7^ or 1.97 × 10^5^ CFU/mouse). The challenge dose was delivered by gavage in 0.2 ml of meat broth culture medium. The mice were then monitored and scored 1 to 3 times daily for weight loss, activity, posture, coat quality, diarrhea, and eye/nose symptoms (56). Mice with a score of 14/20 or above and/or with a ≥20% weight loss were considered at a humane endpoint and were euthanized. Any mouse found dead was given a score of 20. Survivors were followed and euthanized approximately 3 weeks after infection.

### Antibody quantification.

Whole toxin A (List Biologicals, Campbell, CA) or recombinant rbdB was used to coat U-bottom high-binding 96-well ELISA plates (Greiner Bio-One, Frickenhausen, Germany). A standard curve was generated for each plate using mouse IgG antibodies (Sigma-Aldrich) or mouse IgA antibodies (Sigma-Aldrich). The plates were coated with 50 μl of toxin A (1.0 μg/ml), rrbdB (0.25 μg/ml), or IgG/IgA standards overnight at 4°C in 100 mM bicarbonate/carbonate buffer (pH 9.5). The wells were washed with PBS 3 times and then blocked with 150 μl of 2% bovine serum albumin (BSA; Sigma-Aldrich) in PBS–Tween 20 (0.05%; blocking buffer; Fisher Scientific) for 1 h at 37°C. Serum samples were heat inactivated at 56°C for 30 min before dilution 1:50 in blocking buffer. Intestinal supernatants were added to the plates neat. All sample dilutions, including dilutions for the standard curve, were assayed in duplicate (50 μl/well). The plates were incubated for 1 h at 37°C and then washed 4 times with PBS prior to the addition of either horseradish peroxidase (HRP)-conjugated anti-mouse total IgG antibodies (75 μl/well at 1:20,000 in blocking buffer; Sigma-Aldrich) or HRP-conjugated anti-mouse IgA antibodies (75 μl/well at 1:10,000 in blocking buffer; Sigma-Aldrich). The plates were incubated for 30 min (IgG) or 1 h (IgA) at 37°C. Six washes with PBS were performed before the addition of 100 μl/well of 3,3′,5,5′-tetramethylbenzidine (TMB) detection substrate (Millipore, Billerica, MA). Reactions were stopped after 15 min with 50 μl/well of 0.5 M H_2_SO_4_. The plates were read at 450 nm on an EL800 microplate reader (BioTek Instruments Inc., Winooski, VT). The concentration of antigen-specific antibodies in each well (in nanograms per milliliter) was estimated by extrapolation from the standard curve.

### Statistical analysis.

Statistical analysis was performed using GraphPad Prism (version 6) software. For analysis of antibody titers, a one-way nonparametric Kruskal-Wallis analysis of variance was performed with Dunn’s multiple-comparison analysis for comparison of all groups. Statistical significance was considered to have been achieved when *P* was ≤0.05. Data are presented as the means ± standard deviations (SD) or the means ± standard errors of the means (SEM). For analysis of survival, the log-rank (Mantel-Cox) test was used to compare all groups to the PBS control group. The Bonferroni method was used to correct for multiple comparisons. In Table S3, correlations are based on Spearman’s *r* coefficient (nonparametric), 95% confidence intervals were calculated, and two-tailed *P* values were determined.

### Data availability.

The data that support the findings of this study are available from the corresponding author upon reasonable request.

## Supplementary Material

Supplemental file 1

## References

[1] Heimann SM, Cruz Aguilar MR, Mellinghof S, Vehreschild M 2018 Economic burden and cost-effective management of Clostridium difficile infections. Med Mal Infect 48:23–29. doi:10.1016/j.medmal.2017.10.010. 29336929

[2] Rupnik M, Wilcox MH, Gerding DN 2009 Clostridium difficile infection: new developments in epidemiology and pathogenesis. Nat Rev Microbiol 7:526–536. doi:10.1038/nrmicro2164. 19528959

[3] Ananthakrishnan AN 2011 Clostridium difficile infection: epidemiology, risk factors and management. Nat Rev Gastroenterol Hepatol 8:17–26. doi:10.1038/nrgastro.2010.190. 21119612

[4] Carter GP, Rood JI, Lyras D 2010 The role of toxin A and toxin B in Clostridium difficile-associated disease: past and present perspectives. Gut Microbes 1:58–64. doi:10.4161/gmic.1.1.10768. 20664812 PMC2906822

[5] Reigadas E, Alcala L, Marin M, Martin A, Iglesias C, Bouza E 2016 Role of binary toxin in the outcome of Clostridium difficile infection in a non-027 ribotype setting. Epidemiol Infect 144:268–273. doi:10.1017/S095026881500148X. 26119775

[6] Wiegand PN, Nathwani D, Wilcox MH, Stephens J, Shelbaya A, Haider S 2012 Clinical and economic burden of Clostridium difficile infection in Europe: a systematic review of healthcare-facility-acquired infection. J Hosp Infect 81:1–14. doi:10.1016/j.jhin.2012.02.004. 22498638

[7] Zhang S, Palazuelos-Munoz S, Balsells EM, Nair H, Chit A, Kyaw MH 2016 Cost of hospital management of Clostridium difficile infection in United States—a meta-analysis and modelling study. BMC Infect Dis 16:447. doi:10.1186/s12879-016-1786-6. 27562241 PMC5000548

[8] Surawicz CM, Alexander J 2011 Treatment of refractory and recurrent Clostridium difficile infection. Nat Rev Gastroenterol Hepatol 8:330–339. doi:10.1038/nrgastro.2011.59. 21502971

[9] Greenberg RN, Marbury TC, Foglia G, Warny M 2012 Phase I dose finding studies of an adjuvanted Clostridium difficile toxoid vaccine. Vaccine 30:2245–2249. doi:10.1016/j.vaccine.2012.01.065. 22306375

[10] Bruxelle JF, Pechine S, Collignon A 2018 Immunization strategies against Clostridium difficile. Adv Exp Med Biol 1050:197–225. doi:10.1007/978-3-319-72799-8_12. 29383671

[11] Baliban SM, Michael A, Shammassian B, Mudakha S, Khan AS, Cocklin S, Zentner I, Latimer BP, Bouillaut L, Hunter M, Marx P, Sardesai NY, Welles SL, Jacobson JM, Weiner DB, Kutzler MA 2014 An optimized, synthetic DNA vaccine encoding the toxin A and toxin B receptor binding domains of Clostridium difficile induces protective antibody responses in vivo. Infect Immun 82:4080–4091. doi:10.1128/IAI.01950-14. 25024365 PMC4187890

[12] Ibarra JA, Steele-Mortimer O 2009 Salmonella—the ultimate insider. Salmonella virulence factors that modulate intracellular survival. Cell Microbiol 11:1579–1586. doi:10.1111/j.1462-5822.2009.01368.x. 19775254 PMC2774479

[13] Bezay N, Ayad A, Dubischar K, Firbas C, Hochreiter R, Kiermayr S, Kiss I, Pinl F, Jilma B, Westritschnig K 2016 Safety, immunogenicity and dose response of VLA84, a new vaccine candidate against Clostridium difficile, in healthy volunteers. Vaccine 34:2585–2592. doi:10.1016/j.vaccine.2016.03.098. 27079932

[14] Guo S, Yan W, McDonough SP, Lin N, Wu KJ, He H, Xiang H, Yang M, Moreira MA, Chang Y-F 2015 The recombinant Lactococcus lactis oral vaccine induces protection against C. difficile spore challenge in a mouse model. Vaccine 33:1586–1595. doi:10.1016/j.vaccine.2015.02.006. 25698490

[15] Hong HA, Hitri K, Hosseini S, Kotowicz N, Bryan D, Mawas F, Wilkinson AJ, van Broekhoven A, Kearsey J, Cutting SM 2017 Mucosal antibodies to the C terminus of toxin A prevent colonization of Clostridium difficile. Infect Immun 85:e01060-16. doi:10.1128/IAI.01060-16. 28167669 PMC5364299

[16] Makvandi M, Teimoori A, Parsa Nahad M, Khodadadi A, Cheshmeh MGD, Zandi M 2018 Expression of Salmonella typhimurium and Escherichia coli flagellin protein and its functional characterization as an adjuvant. Microb Pathog 118:87–90. doi:10.1016/j.micpath.2018.03.016. 29530809

[17] Ghose C, Verhagen JM, Chen X, Yu J, Huang Y, Chenesseau O, Kelly CPP, Ho DD 2013 Toll-like receptor 5-dependent immunogenicity and protective efficacy of a recombinant fusion protein vaccine containing the nontoxic domains of Clostridium difficile toxins A and B and Salmonella enterica serovar Typhimurium flagellin in a mouse model of Clostridium difficile disease. Infect Immun 81:2190–2196. doi:10.1128/IAI.01074-12. 23545305 PMC3676027

[18] Hayashi F, Smith KD, Ozinsky A, Hawn TR, Yi EC, Goodlett DR, Eng JK, Akira S, Underhill DM, Aderem A 2001 The innate immune response to bacterial flagellin is mediated by Toll-like receptor 5. Nature 410:1099–1103. doi:10.1038/35074106. 11323673

[19] Jepson MA, Clark MA 2001 The role of M cells in Salmonella infection. Microbes Infect 3:1183–1190. doi:10.1016/S1286-4579(01)01478-2. 11755406

[20] Penha Filho RA, Moura BS, de Almeida AM, Montassier HJJ, Barrow PA, Berchieri Junior A 2012 Humoral and cellular immune response generated by different vaccine programs before and after Salmonella Enteritidis challenge in chickens. Vaccine 30:7637–7643. doi:10.1016/j.vaccine.2012.10.020. 23085366

[21] Clark-Curtiss JE, Curtiss R 2018 Salmonella vaccines: conduits for protective antigens. J Immunol 200:39–48. doi:10.4049/jimmunol.1600608. 29255088

[22] Galen JE, Buskirk AD, Tennant SM, Pasetti MF 3 11 2016 Live attenuated human Salmonella vaccine candidates: tracking the pathogen in natural infection and stimulation of host immunity. EcoSal Plus 2016 doi:10.1128/ecosalplus.ESP-0010-2016. PMC5119766 27809955

[23] Panthel K, Meinel KM, Sevil Domènech VEE, Trülzsch K, Rüssmann H 2008 Salmonella type III-mediated heterologous antigen delivery: a versatile oral vaccination strategy to induce cellular immunity against infectious agents and tumors. Int J Med Microbiol 298:99–103. doi:10.1016/j.ijmm.2007.07.002. 17719275

[24] Gerlach RG, Hensel M 2007 Salmonella pathogenicity islands in host specificity, host pathogen-interactions and antibiotics resistance of Salmonella enterica. Berl Munch Tierarztl Wochenschr 120:317–327. 17715824

[25] Xiong G, Husseiny MI, Song L, Erdreich-Epstein A, Shackleford GM, Seeger RC, Jackel D, Hensel M, Metelitsa LS 2010 Novel cancer vaccine based on genes of Salmonella pathogenicity island 2. Int J Cancer 126:2622–2634. doi:10.1002/ijc.24957. 19824039 PMC2993175

[26] Clairmont C, Lee KC, Pike J, Ittensohn M, Low KB, Pawelek J, Bermudes D, Brecher SM, Margitich D, Turnier J, Li Z, Luo X, King I, Zheng LM 2000 Biodistribution and genetic stability of the novel antitumor agent VNP20009, a genetically modified strain of Salmonella typhimurium. J Infect Dis 181:1996–2002. doi:10.1086/315497. 10837181

[27] Toso JF, Gill VJ, Hwu P, Marincola FM, Restifo NP, Schwartzentruber DJ, Sherry RM, Topalian SL, Yang JC, Stock F, Freezer LJ, Morton KE, Seipp C, Haworth L, Mavroukakis S, White D, MacDonald S, Mao J, Sznol M, Rosenberg SA 2002 Phase I study of the intravenous administration of attenuated Salmonella typhimurium to patients with metastatic melanoma. J Clin Oncol 20:142–152. doi:10.1200/JCO.2002.20.1.142. 11773163 PMC2064865

[28] Chen G, Dai Y, Chen J, Wang X, Tang B, Zhu Y, Hua Z 2011 Oral delivery of the Sj23LHD-GST antigen by Salmonella typhimurium type III secretion system protects against Schistosoma japonicum infection in mice. PLoS Negl Trop Dis 5:e1313. doi:10.1371/journal.pntd.0001313. 21909450 PMC3167783

[29] Donald RGK, Flint M, Kalyan N, Johnson E, Witko SE, Kotash C, Zhao P, Megati S, Yurgelonis I, Lee PK, Matsuka YV, Severina E, Deatly A, Sidhu M, Jansen KU, Minton NP, Anderson AS 2013 A novel approach to generate a recombinant toxoid vaccine against Clostridium difficile. Microbiology 159:1254–1266. doi:10.1099/mic.0.066712-0. 23629868 PMC3749728

[30] Tian J-H, Fuhrmann SR, Kluepfel-Stahl S, Carman RJ, Ellingsworth L, Flyer DC 2012 A novel fusion protein containing the receptor binding domains of C. difficile toxin A and toxin B elicits protective immunity against lethal toxin and spore challenge in preclinical efficacy models. Vaccine 30:4249–4258. doi:10.1016/j.vaccine.2012.04.045. 22537987

[31] Wilcox MH, Gerding DN, Poxton IR, Kelly C, Nathan R, Birch T, Cornely OA, Rahav G, Bouza E, Lee C, Jenkin G, Jensen W, Kim Y-S, Yoshida J, Gabryelski L, Pedley A, Eves K, Tipping R, Guris D, Kartsonis N, Dorr M-B, MODIFY I and MODIFY II Investigators. 2017 Bezlotoxumab for prevention of recurrent Clostridium difficile infection. N Engl J Med 376:305–317. doi:10.1056/NEJMoa1602615. 28121498

[32] Kociolek LK, Gerding DN 2016 Breakthroughs in the treatment and prevention of Clostridium difficile infection. Nat Rev Gastroenterol Hepatol 13:150–160. doi:10.1038/nrgastro.2015.220. 26860266

[33] Wang Y, Wang S, Bouillaut L, Li C, Duan Z, Zhang K, Ju X, Tzipori S, Sonenshein AL, Sun X, Young VB 2018 Oral immunization with nontoxigenic Clostridium difficile strains expressing chimeric fragments of TcdA and TcdB elicits protective immunity against C. difficile infection in both mice and hamsters. Infect Immun 86:e00489-18. doi:10.1128/IAI.00489-18. 30150259 PMC6204701

[34] Kardani K, Bolhassani A, Shahbazi S 2016 Prime-boost vaccine strategy against viral infections: mechanisms and benefits. Vaccine 34:413–423. doi:10.1016/j.vaccine.2015.11.062. 26691569

[35] Lakhashe SK, Byrareddy SN, Zhou M, Bachler BC, Hemashettar G, Hu SL, Villinger F, Else JG, Stock S, Lee SJ, Vargas-Inchaustegui DA, Cofano EB, Robert-Guroff M, Johnson WE, Polonis VR, Forthal DN, Loret EP, Rasmussen RA, Ruprecht RM 2014 Multimodality vaccination against clade C SHIV: partial protection against mucosal challenges with a heterologous tier 2 virus. Vaccine 32:6527–6536. doi:10.1016/j.vaccine.2014.08.065. 25245933 PMC4343195

[36] Luke CJ, Subbarao K 2014 Improving pandemic H5N1 influenza vaccines by combining different vaccine platforms. Expert Rev Vaccines 13:873–883. doi:10.1586/14760584.2014.922416. 24855993

[37] Solanki AK, Bhatia B, Kaushik H, Deshmukh SK, Dixit A, Garg LC 2017 Clostridium perfringens beta toxin DNA prime-protein boost elicits enhanced protective immune response in mice. Appl Microbiol Biotechnol 101:5699–5708. doi:10.1007/s00253-017-8333-2. 28523396

[38] Shukarev G, Callendret B, Luhn K, Douoguih M, EBOVAC1 Consortium. 2017 A two-dose heterologous prime-boost vaccine regimen eliciting sustained immune responses to Ebola Zaire could support a preventive strategy for future outbreaks. Hum Vaccin Immunother 13:266–270. doi:10.1080/21645515.2017.1264755. 27925844 PMC5328205

[39] Knudsen ML, Ljungberg K, Kakoulidou M, Kostic L, Hallengärd D, García-Arriaza J, Merits A, Esteban M, Liljeström P 2014 Kinetic and phenotypic analysis of CD8^+^ T cell responses after priming with alphavirus replicons and homologous or heterologous booster immunizations. J Virol 88:12438–12451. doi:10.1128/JVI.02223-14. 25122792 PMC4248943

[40] Best EL, Freeman J, Wilcox MH 2012 Models for the study of Clostridium difficile infection. Gut Microbes 3:145–167. doi:10.4161/gmic.19526. 22555466 PMC3370947

[41] Cohen OR, Steele JA, Zhang Q, Schmidt DJ, Wang Y, Hamel PE, Beamer G, Xu B, Tzipori S 2014 Systemically administered IgG anti-toxin antibodies protect the colonic mucosa during infection with Clostridium difficile in the piglet model. PLoS One 9:e111075. doi:10.1371/journal.pone.0111075. 25347821 PMC4210241

[42] van Kleef E, Deeny SR, Jit M, Cookson B, Goldenberg SD, Edmunds WJ, Robotham JV 2016 The projected effectiveness of Clostridium difficile vaccination as part of an integrated infection control strategy. Vaccine 34:5562–5570. doi:10.1016/j.vaccine.2016.09.046. 27727031

[43] Prisco A, De Berardinis P 2012 Memory immune response: a major challenge in vaccination. Biomol Concepts 3:479–486. doi:10.1515/bmc-2012-0010. 25436552

[44] Haselbeck AH, Panzner U, Im J, Baker S, Meyer CG, Marks F 2017 Current perspectives on invasive nontyphoidal Salmonella disease. Curr Opin Infect Dis 30:498–503. doi:10.1097/QCO.0000000000000398. 28731899 PMC7680934

[45] Karsten V, Murray SR, Pike J, Troy K, Ittensohn M, Kondradzhyan M, Low KB, Bermudes D 2009 msbB deletion confers acute sensitivity to CO_2_ in Salmonella enterica serovar Typhimurium that can be suppressed by a loss-of-function mutation in zwf. BMC Microbiol 9:170. doi:10.1186/1471-2180-9-170. 19689794 PMC2745414

[46] Hindle Z, Chatfield SN, Phillimore J, Bentley M, Johnson J, Cosgrove CA, Ghaem-Maghami M, Sexton A, Khan M, Brennan FR, Everest P, Wu T, Pickard D, Holden DW, Dougan G, Griffin GE, House D, Santangelo JD, Khan SA, Shea JE, Feldman RG, Lewis DJ 2002 Characterization of Salmonella enterica derivatives harboring defined aroC and Salmonella pathogenicity island 2 type III secretion system (ssaV) mutations by immunization of healthy volunteers. Infect Immun 70:3457–3467. doi:10.1128/iai.70.7.3457-3467.2002. 12065485 PMC128087

[47] Yen C, Jakob K, Esona MD, Peckham X, Rausch J, Hull JJ, Whittier S, Gentsch JR, LaRussa P 2011 Detection of fecal shedding of rotavirus vaccine in infants following their first dose of pentavalent rotavirus vaccine. Vaccine 29:4151–4155. doi:10.1016/j.vaccine.2011.03.074. 21477676 PMC4459210

[48] Rota PA, Khan AS, Durigon E, Yuran T, Villamarzo YS, Bellini WJ 1995 Detection of measles virus RNA in urine specimens from vaccine recipients. J Clin Microbiol 33:2485–2488. 7494055 10.1128/jcm.33.9.2485-2488.1995 PMC228449

[49] Troy SB, Ferreyra-Reyes L, Huang C, Sarnquist C, Canizales-Quintero S, Nelson C, Báez-Saldaña R, Holubar M, Ferreira-Guerrero E, García-García L, Maldonado YA 2014 Community circulation patterns of oral polio vaccine serotypes 1, 2, and 3 after Mexican national immunization weeks. J Infect Dis 209:1693–1699. doi:10.1093/infdis/jit831. 24367038 PMC4017366

[50] Freer G, Pistello M 2018 Varicella-zoster virus infection: natural history, clinical manifestations, immunity and current and future vaccination strategies. New Microbiol 41:95–105. 29498740

[51] Santos RL, Zhang S, Tsolis RM, Kingsley RA, Adams LG, Baumler AJ 2001 Animal models of Salmonella infections: enteritis versus typhoid fever. Microbes Infect 3:1335–1344. doi:10.1016/S1286-4579(01)01495-2. 11755423

[52] Carter GP, Chakravorty A, Pham Nguyen TA, Mileto S, Schreiber F, Li L, Howarth P, Clare S, Cunningham B, Sambol SP, Cheknis A, Figueroa I, Johnson S, Gerding D, Rood JI, Dougan G, Lawley TD, Lyras D 2015 Defining the roles of TcdA and TcdB in localized gastrointestinal disease, systemic organ damage, and the host response during Clostridium difficile infections. mBio 6:e00551-15. doi:10.1128/mBio.00551-15. 26037121 PMC4453007

[53] Kuehne SA, Cartman ST, Heap JT, Kelly ML, Cockayne A, Minton NP 2010 The role of toxin A and toxin B in Clostridium difficile infection. Nature 467:711–713. doi:10.1038/nature09397. 20844489

[54] Avezov E, Cross BC, Kaminski Schierle GS, Winters M, Harding HP, Melo EP, Kaminski CF, Ron D 2013 Lifetime imaging of a fluorescent protein sensor reveals surprising stability of ER thiol redox. J Cell Biol 201:337–349. doi:10.1083/jcb.201211155. 23589496 PMC3628511

[55] Chen X, Katchar K, Goldsmith JD, Nanthakumar N, Cheknis A, Gerding DN, Kelly CP 2008 A mouse model of Clostridium difficile-associated disease. Gastroenterology 135:1984–1992. doi:10.1053/j.gastro.2008.09.002. 18848941

[56] Warren CA, van Opstal E, Ballard TE, Kennedy A, Wang X, Riggins M, Olekhnovich I, Warthan M, Kolling GL, Guerrant RL, Macdonald TL, Hoffman PS 2012 Amixicile, a novel inhibitor of pyruvate:ferredoxin oxidoreductase, shows efficacy against Clostridium difficile in a mouse infection model. Antimicrob Agents Chemother 56:4103–4111. doi:10.1128/AAC.00360-12. 22585229 PMC3421617

