# Supplementary material for: Vaccination against Clostridium difficile by Use of an Attenuated Salmonella enterica Serovar Typhimurium Vector (YS1646) Protects Mice from Lethal Challenge
Source: Infect Immun. 2019 Jul 23;87(8):e00089-19. doi: 10.1128/IAI.00089-19 (PMC6652760; doi:10.1128/IAI.00089-19)
Supplement: Supplemental file 1 [file IAI.00089-19-s0001.pdf]

Supplemental Table 1

Primers used in Plasmid Construction

	Forward Primer (5' --> 3')	Reverse Primer (5' --> 3')	Source
SopE2 promoter and secretory signal	CCGCTCGAGTAAAAATGTTCCTCG ATAAA	CATGGTAGTTCTCCTTTTAG	YS1646
SptP promoter and secretory signal	CGCCTCGAGTTTACGCTGACTCAT TGG	CATTTTTCTCTCCTCATACTTTA	YS1646
SseJ promoter and secretory signal	CGCCTCGAGACATAAAACACTAGC ACT	CGCCTCGAGACATAAAACACTAG CACT	YS1646
SspH1 promoter and secretory signal	CGCCTCGAGCGCTATATCACCAAA AC	CTCTGCGGCCGCGGTAAGACCTG ACGCTC	YS1646
SspH2 promoter and secretory signal	CGCCTCGAGGTTTGTGCGTCGTAT	CTCTGCGGCCGCATTCAGGCAGG CACGCA	YS1646
SteA promoter and secretory signal	CGCCTCGAGGTTTCGCCGCATGTT G	CTCTGCGGCCGCATAATTGTCCA AATAGT	YS1646
SteB promoter and secretory signal	CGCCTCGAGCGCTCCAGCGCTTCG A	CTCTGCGGCCGCTCTGACATTAC CATTT	YS1646
Lac promoter	CGCCTCGAGCATTAGGCACCCCAG GCTTTACACTTTATGCTTCCGGCTC GTATGTTGTGTGGAATTGTGAGCG GATAA, GTGGAATTGTGAGCGGATAACAAT TTCACACAGGAAACAGCTATGACC ATGACTAACATAACACTATCCAC		Sequence is in the primers
nirB promoter	CGCCTCGAGTTGTGGTTACCGGCC CGAT	CGCGCGGCCGCCGGATCTTTACT CGCATTAC	DH5α *E. coli*
pagC promoter	CGCCTCGAGGTTAACCACTCTTAA TAA	AACAACTCCT TAATACTACT	YS1646
SopE2 Secretion Signal	GGCGGTAATAGAAAAGAAATCGA GGCAAAAATGACTAACATAACACT ATCCAC	AAGTCGCGGCCGCCGGATCTTTA CTCGC	YS1646
SspH1 Secretion Signal	GGCGGTAATAGAAAAGAAATCGA GGCAAAAATGTTTAATATCCGCAA TACACAACCTT	CTCTGCGGCCGCGGTAAGACCTG ACGCTC	YS1646
rbdA	CGCGCGGCCGCGACTTATTACTAT GAT	TAGTCGGCGCGCCCGCCATATAT CCCAGG	VPI 10463
rbdB	CCGGCGGCCGCAGAGAAATTTTAT ATTAAT	AGTCGGCGCGCCGTTCACTAATC ACTAATTG	VPI 10463
EGFP	CGCGCGGCCGCGGTGAGCAAGGG CGAG	AGTCGGCGCGCCTTACTTGTACA GCTCGTC	pEGFP_C1

Primers used to replicate the sequences from source DNA are shown. Some sequences were further edited to include an ATG start site between the promoter and secretory signal.

Supplemental Table 2

***In vitro* and *in vivo* screening of plasmids**

	In vitro						*In vivo* (IM Prime, PO Boost)			
	EGFP Detection (EGFP expressing strains)		Antigen Detection by WB				Serum IgG		Intestinal IgA	
**Strains**	LB	RAW 264.7 (24h)	LB	Secretion in LB	RAW 264.7 (1hr)	RAW 264.7 (24h)	rbdB	rbdA	rbdB	rbdA
pQE_null	0	0	0	0	0	0	0	0	0	0
SopE2_SopE2_rbdB	+++	+++	+	0	0	0	0	<ctl	0	<ctl
SseJ_SseJ_rbdB	+	++	+	0	0	0	++	<ctl	0	0
SptP_SptP_rbdB	+	+	+	0	+	0	+	<ctl	+	0
SspH1_SspH1_rbdB	+	+	0	0	0	0	++	<ctl	++	0
SspH2_SspH2_rbdB	++	++	0	0	0	0	+++	<ctl	+++	0
SteA_SteA_rbdB	+++	++	+	0	0	0	++	<ctl	+++	0
SteB_SteB_rbdB	+	+++	0	0	0	0	<ctl	<ctl	0	0
pagC_SspH1_rbdB	n/a	+++	+	n/a	+	0	n/a	n/a	n/a	n/a
SspH2_SspH2_rbdA	++	++	0	n/a	0	0	n/a	n/a	n/a	n/a
lac_SopE2_rbdA	+	+	+	0	0	0	<ctl	<ctl	0	0
lac_SspH1_rbdA	0	0	0	0	0	0	<ctl	<ctl	0	0
nirB_SopE2_rbdA	++	+	+	0	+	0	<ctl	<ctl	0	++
nirB_SspH1_rbdA	++	+++	+	0	+	0	<ctl	<ctl	0	0
pagC_SopE2_rbdA	n/a	++	0	0	0	0	<ctl	<ctl	0	+++
pagC_SspH1_rbdA	n/a	+++	+	+	+	0	<ctl	<ctl	0	+++

EGFP detection is based on the EGFP expressing strains with the same promoter and secretory signal as the listed strain. Strains that were not assessed are indicated in the table as "n/a". Detection by Western blot is designated as either antigen is detected "+" or not "0". For *in vivo* screening, mice were vaccinated with 10ug of protein IM (rbdA/rbdB) adjuvanted with alum and three weeks later the response was boosted by the YS1646 strains given by PO in 3 doses (n=2-4 mice/group). Serum and intestines were collected 3 weeks after the boost. Titers are shown compared to the control group of the listed protein delivered IM, boosted with pQE_null strain of YS1646. Titers lower than the control are listed as "<ctl". Titers that match the control are listed as "0". Titers higher than the control were divided into three categories; "+", "++", "+++" with increasing mean titers.

Supplemental Table 3

Correlations between antibody titers and clinical scores

		Tox A IgG Pre	Tox A IgG Post	Tox A IgA Post	rbdB IgG Pre	rbdB IgG Post	rbdB IgA Post	Salmonella IgG Pre	Salmonella IgG Post
Mean Score (all)	p value	ns	ns	ns	****	****	**	*	ns
	r	/	/	/	-0.735	-0.6555	-0.5047	-0.4031	/
	95% CI	/	/	/	(-0.8554, -0.5392)	(-0.8189, -0.3939)	(-0.7278, -0.1849)	(-0.6433, -0.09067)	/
Mean Score (vax)	p value	ns	ns	ns	**	**	**	/	/
	r	/	/	/	-0.7191	-0.6744	-0.6708	/	/
	95% CI	/	/	/	(-0.9031, -0.3124)	(-0.8857, -0.2318)	(-0.8843, -0.2257)	/	/
Highest Score (all)	p value	ns	ns	ns	****	***	**	*	ns
	r	/	/	/	-0.7177	-0.6068	-0.5158	-0.4031	/
	95% CI	/	/	/	(-0.8453, -0.5128)	(-0.7904, -0.3234)	(-0.7348, -0.1994)	(-0.6433, -0.09067)	/
Highest Score (vax)	p value	ns	*	*	ns	ns	*	*	/
	r	/	0.5643	0.6453	/	/	-0.6238	-0.3741	/
	95% CI	/	(0.0008807, 0.8558)	(0.1283, 0.8865)	/	/	(-0.8653, -0.1475)	(-0.6288, -0.05671)	/

Correlations are based on Spearman's r coefficient (non-parametric), 95% Confidence Intervals were calculated, and a two tailed p value was determined. * $P<0.05$, ** $P<0.01$, *** $P<0.001$, **** $P<0.0001$

Supplemental Figure 1

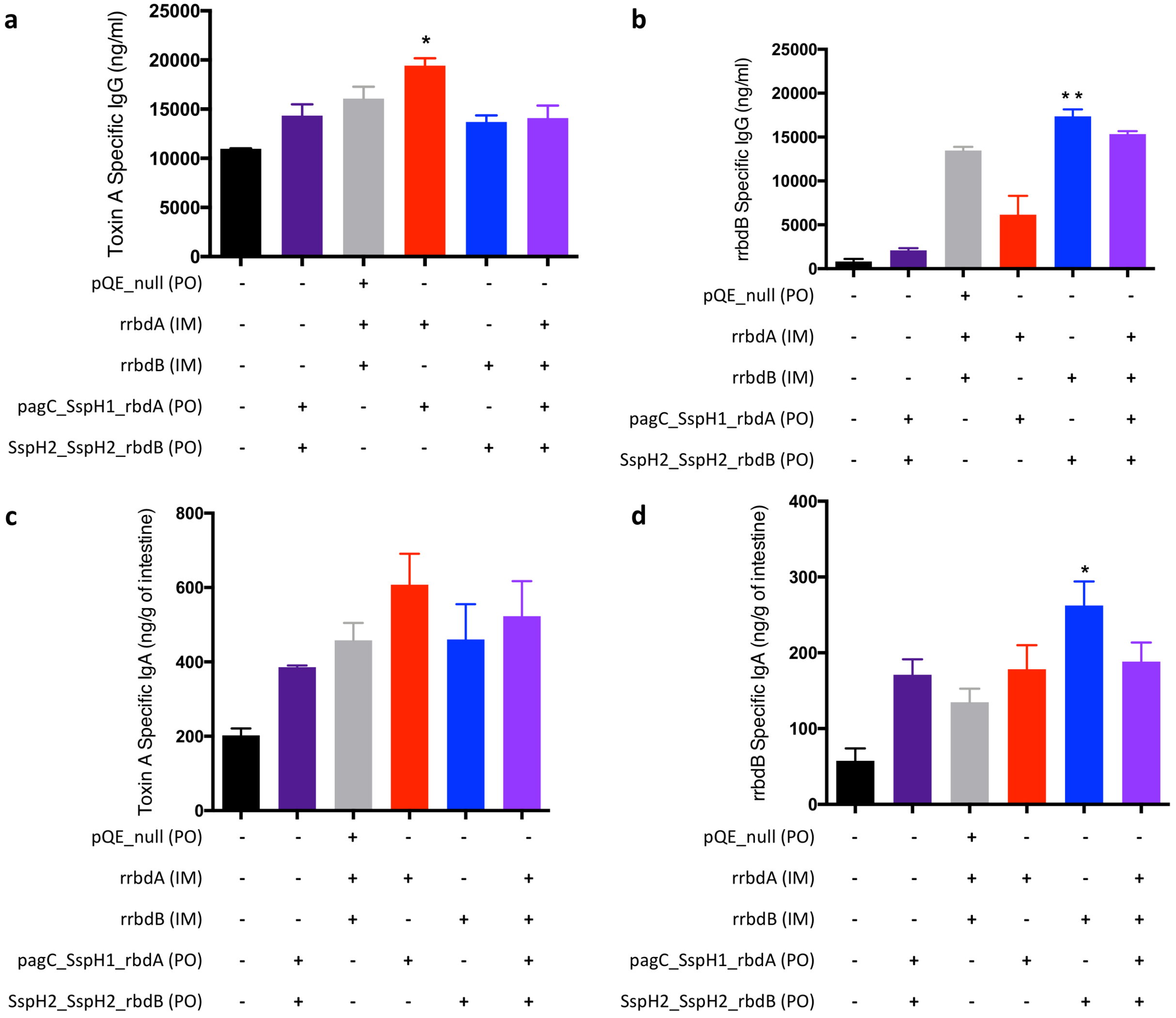

Vaccination with antigen expressing YS1646 increases post-challenge antibody titers in the sera and intestines of survivors. Mice were immunised with a dose of 10µg recombinant antigen (rrbdA and/or rrbdB) intramuscularly, and three doses of 1×10^9 cfu of antigen expressing YS1646 (pagC_SspH1_rbdA and/or SspH2_SspH2_rbdB), orally every other day. 5 weeks after vaccination, mice were challenged with 1.7×10^7 cfu of *C. difficile*. 3 weeks after infection, serum and intestines were collected from survivors. Post-challenge serum toxin A specific IgG antibodies (**a**) and rrbdB specific IgG antibodies (**b**) were detected by ELISA. Intestinal toxin A specific IgA antibodies (**c**) and rrbdB specific IgA antibodies (**d**) were detected by ELISA (n= 2-8, one experiment). Mean and standard error of the mean (SEM) are shown. Kruskal-Wallis test and Dunn's Multiple Comparison test were used to compare between all groups. * P<0.05, ** P<0.01, *** P<0.001, **** P<0.0001 compared to the PBS control group.
